# The Sequel in Cases of Pylorectomy

**Published:** 1902-01-18

**Authors:** 


[1] Lancet, Jan. 11.

### THE SEQUEL IN CASES OF PYLORECTOMY.

It is always a matter of interest to know the end of cases, especially when serious operations have been performed. Dr. J. Rutherford Morison [1] relates the after history of five cases in which partial gastrectomy had been performed for cancer of the pylorus. The first case was a woman aged 40 years. Pylorectomy was performed on October 21st, 1897. She was perfectly well until May, 1900, being able to eat and digest any ordinary food and to do her household duties. In May, 1900, she began to have uneasy sensations after food, with pyrosis, and she discovered a lump in her epigastrium. On July 24th her abdomen was opened and a large adherent tumour was discovered. The abdomen was closed. She got worse and died at home in December, 1900, three years and two months after the first operation. The second case was a male aged 48 years, with dyspeptic symptoms and a hard, nodulated tumour. Pylorectomy was performed on September 12th, 1898. He greatly improved, and a year later looked in good health, and was able to eat and digest ordinary food, and was doing a full day's work. In February, 1900, he began not to feel so well, and to lose flesh, and a small skin nodule was found immediately to the right of the scar. By the end of August there was a large mass, and he died on November 3rd, 1900, two years and a half after the operation. Post-mortem examination showed that there was no sign of growth in the stomach itself, but the glands were enlarged and infiltrated, and the liver was studded throughout with large masses of growth. The third case was a woman aged 41 years. Pylorectomy was performed on September 19th, 1898. The patient quickly improved, gained flesh, and was able to work until May, 1899, when stomach symptoms returned, and in July a hard mass was found. She died in December, 1899, one year and three months after the operation. The fourth case was a man aged 38 years. The tumour was the size of a cocoanut, and moved freely on respiration. Pylorectomy was performed on October 18th, 1898. In September, 1899, the patient returned and reported himself in perfect health. Again, in 1900, he wrote to say that he was perfectly well and was still gaining flesh. In March, 1901, he was perfectly well, was able to eat anything and was doing his work. But he had lost four pounds in weight since the preceding September. This seems to have been the beginning of a downhill course, and in July there were signs of a large local recurrence with deposits in the liver. He died at home on September 19, 1901, two years and eleven months after the operation. The fifth case was a man aged 41 years, who had been ill for five months and had been vomiting blood. Pylorectomy was performed on February 9, 1899. After the operation he gained flesh and became able to eat ordinary food, but he gradually got worse again and died at home about six months after the operation. A most interesting series of cases. There seems every reason to believe that if the disease could be discovered early enough it might be completely removed. It is the old, old lesson, the disease not being discovered until too late. The symptoms appear to have existed for five months, two months, fourteen months, six weeks, and five months respectively, in the several cases, before they came under treatment.

